# Humanized Chronic Graft-versus-Host Disease in NOD-SCID il2rγ-/- (NSG) Mice with G-CSF-Mobilized Peripheral Blood Mononuclear Cells following Cyclophosphamide and Total Body Irradiation

**DOI:** 10.1371/journal.pone.0133216

**Published:** 2015-07-15

**Authors:** Hisaki Fujii, Zhi-Juan Luo, Hye Jin Kim, Susan Newbigging, Adam Gassas, Armand Keating, R. Maarten Egeler

**Affiliations:** 1 Hematology/Oncology/BMT, The Hospital for Sick Children, Toronto, ON, Canada; 2 Developmental Stem Cell Biology, The Hospital for Sick Children, Toronto, ON, Canada; 3 Toronto Centre for Phenogenomics, Toronto, ON, Canada; 4 Princess Margaret Hospital, Toronto, ON, Canada; University of California, San Francisco, UNITED STATES

## Abstract

Chronic graft-versus-host disease (cGvHD) is the major source of late phase morbidity and mortality after allogeneic hematopoietic stem cell transplantation. Humanized acute GvHD (aGvHD) *in vivo* models using NOD-SCID il2rγ-/- (NSG) mice are well described and are important tools for investigating pathogenicity of human cells *in vivo*. However, there have been only few reported humanized cGvHD mouse models. We evaluated if prolonged inflammation driven by low dose G-CSF-mobilized human PBMCs (G-hPBMCs) would lead to cGvHD following cyclophosphamide (CTX) administration and total body irradiation (TBI) in NSG mice. Engraftment was assessed in peripheral blood (PB) and in specific target organs by either flow cytometry or immunohistochemistry (IHC). Tissue samples were harvested 56 days post transplantation and were evaluated by a pathologist. Some mice were kept for up to 84 days to evaluate the degree of fibrosis. Mice that received CTX at 20mg/kg did not show aGvHD with stable expansion of human CD45^+^ CD3^+^ T-cells in PB (mean; 5.8 to 23.2%). The pathology and fibrosis scores in the lung and the liver were significantly increased with aggregation of T-cells and hCD68^+^ macrophages. There was a correlation between liver pathology score and the percentage of hCD68^+^ cells, suggesting the role of macrophage in fibrogenesis in NSG mice. In order to study long-term survival, 6/9 mice who survived more than 56 days showed increased fibrosis in the lung and liver at the endpoint, which suggests the infiltrating hCD68^+^ macrophages may be pathogenic. It was shown that the combination of CTX and TBI with a low number of G-hPBMCs (1x10^6^) leads to chronic lung and liver inflammation driven by a high infiltration of human macrophage and mature human T cells from the graft, resulting in fibrosis of lung and liver in NSG mice. In conclusion this model may serve as an important pre-clinical model to further current understanding of the roles of human macrophages in cGvHD.

## Introduction

Allogeneic hematopoietic stem cell transplantation (allo-HSCT) is widely used for the treatment of hematological malignancies and other disorders including immune deficiencies, inborn errors of metabolism and hemoglobinopathies. Graft-versus-Host Disease (GvHD) is the major source of morbidity and mortality following allo-HSCT. GvHD is typically classified as either acute or chronic largely depending on the length of time before disease onset and/or clinical manifestations[1]. Chronic GvHD (cGvHD) occurs in 40%-70% of allo-HSCT patients. The quality of life of these patients is severely affected, and the two-year overall survival is only 62%[2]. cGvHD presents with autoimmune-like and fibrotic symptoms, and B cell activation appears to be involved in this process[3,4]. According to the NIH global severity score[2], the skin, mouth, eyes and liver are the most commonly involved organs in moderate to severe cGvHD, while the lung is considered to have the most severe form of cGvHD. Currently, corticosteroids are used as the standard treatment for cGvHD with limited success. However, at this point, there are no effective second line therapies for cGvHD[5].

To develop a new treatment for this complicated disease, an *in vivo* system that recapitulates the human pathophysiology is required. Mouse cGvHD models have been used in investigating mechanism of cGvHD[6], however most of the models exhibit only skin or kidney damage. No mouse model thus far has recapitulated all of the factors contributing to cGvHD pathophysiology such as: defective negative selection due to thymic damage, reduced regulatory T-cell numbers, increased fibrogenic cytokines and activated autoreactive B cells.

Mice with a humanized immune system have been developed to investigate the function of human hematopoietic cells *in vivo* [7,8]. NOD/SCID IL2γchain^-/-^ (NSG) mice, that lack T, B, natural killer (NK) and dendritic cells, are most widely used due to high engraftment of human cells. NSG mice bearing human peripheral blood mononuclear cells (PBMCs) has been shown to develop xenogeneic aGvHD that mimics manifestations of human aGvHD[9–12]. This allows the investigation of the role of human T-cells in mediating xenogeneic GvHD. Therefore, these mice are a strong pre-clinical *in vivo* model for evaluating new treatments including cell therapy products before translation into the clinic.

NOD/SCID or NSG mice transplanted with human bone marrow (BM), liver and thymus (BLT) and fetal liver CD34^+^ cells display hCD4^+^ T cell-mediated scleroderma[13]. Lockrige *et al*. developed a BLT cGvHD mouse model transplanted with hCD34^+^ cells that showed multiple organ involvements more than 120 days post transplantation[14]. This model mimics the multiple organ damage seen in cGvHD.

In this study, we aimed to establish a humanized cGvHD model in order to facilitate discovery of new treatment strategies. Given the history of aGvHD and that Granulocyte Colony Stimulating Factor (G-CSF)-mobilized human peripheral blood mononuclear cells (G-hPBMCs) are known risk factors for cGvHD[15], we tested whether G-hPBMCs with a modified conditioning regimen can give sub-acute inflammation leading to lasting GvHD with cGvHD manifestations in the humanized mouse system.

## Materials and Methods

### Mice

NSG mice were bred and housed under specific pathogen-free conditions in micro-isolators in the animal facility at the Ontario Cancer Institute Animal Resource Center. Experimental procedures were approved by the Animal Care Committee of the University Health Network, and carried out in accordance with the Canadian Council on Animal Care Guidelines. Eight to twelve week-old female mice were used as transplant recipients. The endpoint for the survival study was set when recipient mice looked clinically ill or lost more than 25% of body weight, and mice were humanely sacrificed by cervical dislocation under general anesthetic or CO2.

### Hematopoietic stem cell transplantation

G-CSF-mobilized human peripheral blood mononuclear cells (G-hPBMCs; 3 donors) and CD34^+^ cells from G-CSF-mobilized human PB were purchased from ALLCELLS (Emeryvill, CA) and were stored in liquid nitrogen until use. NSG mice were conditioned with CTX (Sigma Aldrich) by intraperitoneal injection at different doses (20-60mg/kg) on day -3 and -2. A single dose of total body irradiation (TBI) at 200cGy (Precision X-RAD 320 Biological Irradiator) was given on day -1, followed by intravenous injection of 1-5x10^6^ G-hPBMCs or 1x10^5^ CD34^+^ stem cells into the tail vein on day 0. Mice were monitored for weight and clinical symptoms as previously described twice per week[16]. Eight experiments were performed using 3 different donors.

### Histopathology

Samples of skin, lung, liver, spleen and small/large intestine were examined on day 56 or at the time of death post transplantation. These samples were fixed with 10% formalin, embedded in paraffin, and stained with Hematoxylin and Eosin (H&E) and Masson’s trichrome. Disease was quantified blindly by one of the investigators (SN) using a modified cGvHD pathology scale as previously described[17]. Twenty parameters for lung, seventeen for liver and two for spleen were scored from 0 to 4 (S1 Table). Trichrome-stained slides were digitized with a ScanScope (Aperio, Vista, CA). Collagen depositions were quantified on at least 3 different randomly selected sections in the alveolar area of the lung and the whole liver (magnification 20x) as a ratio of blue staining area to the total staining area using Aperio ImageScope. For some experiments, blocks were stained with anti-human CD4 (Ventana prediluted), anti-human CD8 (DAKO, 1:20), anti-human CD20 (Ventana prediluted), anti-human CD68 (Ventana prediluted) and anti-human FoxP3 (eBioscience 1:20) using a Ventana Ultraview DAB detection kit in a Ventana BenchMark XT processor (Ventana, Tucson, AZ).

### Quantitative real time PCR

Total RNA was extracted from lung and liver using an RNeasy kit according to the manufacture protocol (Qiagen), and cDNA was synthesized with equal amounts of total RNA using Superscript VILO cDNA Synthesis Kits. qRT-PCR was performed using SYBR Green PCR master mixture and an ABI ViiA 7 Real-Time PCR System (Life Technologies). All primers for mouse type I collagen (Col1a1), mouse type III collagen (Col3a1), mouse matrix metallopeptidase 3 (MMP3), 9 (MMP9) and mouse Actb were obtained from Integrated DNA technologies (Coralville, IA, USA). The quantity of cDNA for each RNA sample was normalized to the quantity of Actb cDNA in each sample and relative expression was determined using the 2^−ΔCt^ method.

### Flow cytometric analysis

Peripheral blood was collected via saphenous vein using heparin-coated capillary tubes, and was examined for engraftment of human lymphocytes. Erythrocytes were removed by RBC Lysis Buffer (BioLegend, San Diego, CA). Cells were washed with PBS (containing 2% fetal bovine serum [FBS] and 2.5 mM EDTA) and stained for 30 minutes at 4°C using the following mouse monoclonal antibodies: anti-human CD3 (clone HIT3a), CD19 (clone HIB19), CD41 (clone HIP8), anti-mouse CD41 (clone MWReg30) (BioLegend, San Diego, CA), anti-human CD45 (clone HI30) and anti-mouse CD45.1 (clone A20, BD Biosciences, San Jose, CA). Data was acquired on an FC500 (Beckman Coulter) or LSRII (BD Biosciences, San Jose, CA) and was analyzed using FlowJo software (Treestar, Ashland, OR, USA). Donor cells were stained for zombie aqua, anti-human CD3 (BioLegend, San Diego, CA), Annexin V FITC and mouse monoclonal anti-human CD34 (clone 581, BD Biosciences, San Jose, CA) to assess the viability according to the manufacturer’s protocol.

### Statistics

Survival curves were plotted using Kaplan-Meyer estimates. Nonparametric Mann-Whitney *U* tests were used to analyze the significance of all experimental data. All results are presented as mean ± standard deviation (SD). Descriptive statistics were generated on all data using Prism version 6 for Mac (GraphPad Software, San Diego, CA).

## Results

### Low dose of human PBMCs does not cause aGvHD with CTX/TBI

In order to increase the possibility of development of cGvHD, G-hPBMCs were applied as the donor source because of the known high risk of leading to cGvHD in humans[18]. To determine the required number of G-hPBMCs to give rise to aGvHD in NSG mice, mice were infused with G-hPBMCs on day 0 at either 20x10^6^, 10x10^6^, 5x10^6^ or 1x10^6^ cells/mouse following TBI (200cGy). As expected, NSG mice exhibited signs of aGvHD (hunching, weight loss, ruffling hair, reduced mobility) when 5x10^6^ G-hPBMCs or more were infused. Survival rates of mice 56 days post transplantation were as follows; 0/5 (20x10^6^), 4/5 (10x10^6^), 4/6 (5x10^6^), 8/8 (1x10^6^), 8/8 (irradiation only) (S1 Fig).

**Fig 1:**
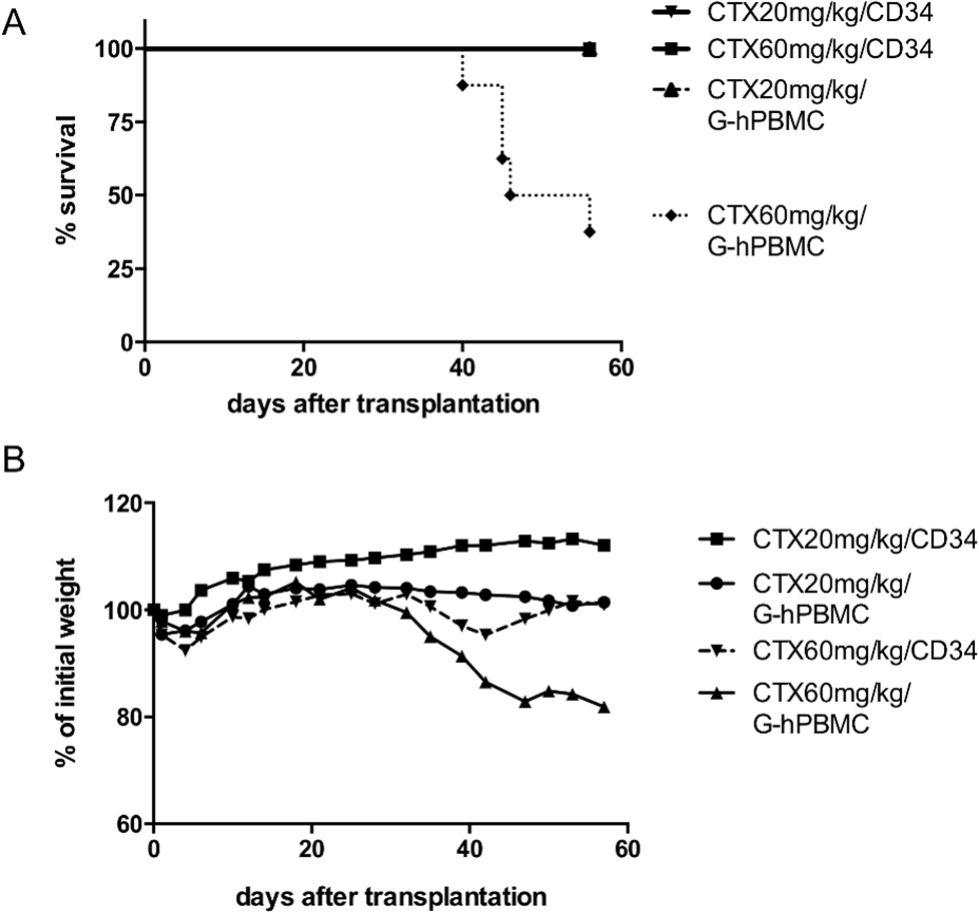
Effect of cyclophosphamide in NSG mice 56 days post transplantation. NSG mice received 20mg/kg or 60mg/kg cyclophosphamide by intraperitoneal injection (day -3, -2) and 200cGy X-ray (day-1) followed by i.v. injection of 1x10^6^ G-hPBMCs or 1x10^5^ CD34^+^ cells on day 0. Survival (A) and weight loss (B) were monitored until 56 days post transplantation. Each experiment was done in triplicate and every group consisted of at least 7 mice.

Next, the dose effect of CTX combined with TBI was evaluated *in vivo*. NSG mice were given CTX at either 0mg/kg, 20mg/kg or 60mg/kg per dose per day for 2 days followed by 200cGy TBI and 1x10^6^ G-hPBMC infusion. As shown in Fig 1B, mice that received CTX 60mg/kg lost weight regardless of the stem cell source, suggesting that CTX was too toxic at this dose. However, mice that received 20mg/kg CTX did not show any sign of aGvHD such as acute illness, weight loss or diarrhea and survived for more than 8 weeks post transplantation (Fig 1A).

**Fig 2:**
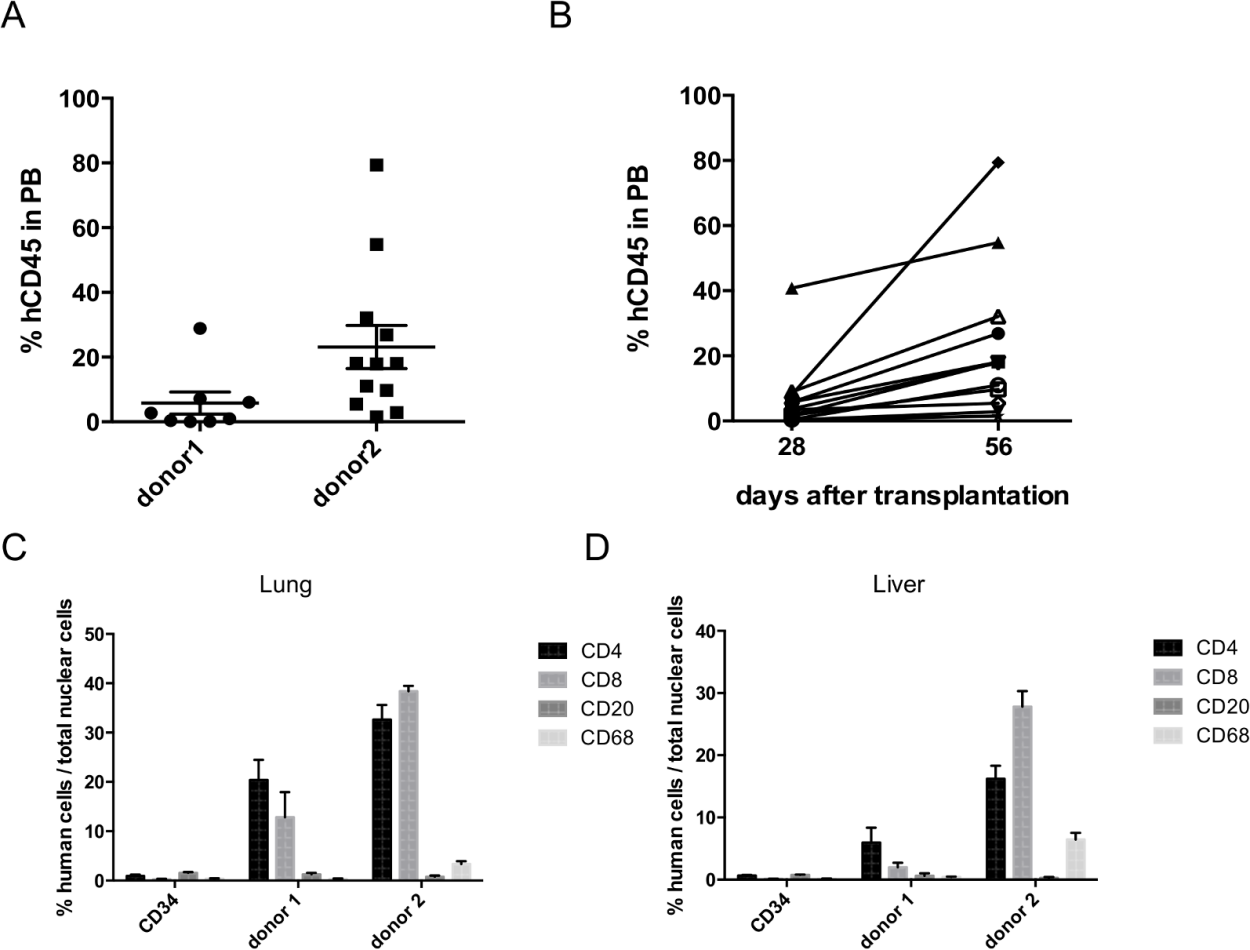
Engraftment of human hematopoietic cells in NSG mice. Peripheral blood chimerism analysis (% of hCD45^+^ cells) was performed by flow cytometry 56 days post transplantation. (A; n = 8 donor 1, n = 12 donor 2). In donor 2, % of hCD3/hCD45^+^ cells was compared on day 28 and day 56 (B; n = 12 mice each). Lung (C) or liver (D) from NSG mice were examined for human CD4, CD8, CD20 and CD68 engraftment by IHC 56 days post transplantation. Three randomly selected sections per slide from at least 3 mice each were placed on the optical photomicroscope and observed under the X20 objective. Data were calculated as: % positive cells = positive nuclei cells ⁄ total cells nuclei x 100. All results are presented as mean ± SEM.

To ensure that 20mg/kg CTX and 200cGy TBI followed by infusion of 1x10^6^ G-hPBMCs could achieve durable engraftment, the percentages of hCD45^+^ cells in PB were evaluated by flow cytometry 56 days post transplantation. Mice that received 1x10^6^ G-hPBMCs had a mean of 5.8±3.4(SE) % hCD45^+^ cells (donor 1) and 23.2±6.7(SE) % (donor 2) in PB respectively (Fig 2A), and mostly consisted of hCD3^+^ T cells (>95%). G-hPBMC at a dose of 1x10^6^ from donor 3 showed little or no engraftment at day 56. To explain the difference among donors, percentages of apoptosis in CD3^+^ and CD34^+^ cell populations before *in vivo* administration were examined by flow cytometry. Donor 2 had the lowest percentage of CD3^+^ T-cells in the graft (27.0% of lymphocytes) with CD3^+^ T-cell viability of 59.5% (Annexin V^-^/ Fixable Aqua^-^) followed by donor 3 (percentage of CD3^+^ T cells 29.2%, viability 43.5%) and donor 1 (percentage of CD3+ T cell 42.2%, viability 53.0%). Donor 2 had the highest percentage of CD34^+^ cells (2.58%) and these were 88.9% viable. Donor 3, had 1.34% CD34^+^ cells (87.9% viable) and donor 1 had 0.86% CD34^+^ cells (87.2% viable) (S2 Fig). Since donor 3 had little engraftment, the absolute number of infused viable CD3^+^T and CD34^+^ cells in one million G-hPBMC was investigated. As expected, donor 3 had the lowest number of CD3^+^T cells (1.3x10^5^) followed by donor 2 (1.7x10^5^) and donor 1 (2.5x10^5^). Similar to the percentage, donor 2 had the highest absolute number of CD34^+^ cells (2.2x10^4^) followed by donor 3 (1.1x10^4^) and donor 1 (0.74x10^4^). Due to low viability/number of CD3^+^ cells, donor 3 was excluded from the initial analysis. In donor 2, we examined the kinetics of engraftment at day 28 and 56 post transplantation. As shown in Fig 2B, all mice achieved higher engraftment of hCD45^+^ cells at day 56 compared to day 28 post transplantation. To confirm hematopoietic recovery from hematopoietic stem cells, platelet recovery was examined by flow cytometry in mice with G-hPBMCs. All mice (12/12) showed human platelet recovery in PB (S3 Fig), which demonstrates the engraftment of CD34^+^ cells in the model.

### Engraftment of human T cells/macrophages in tissues post transplantation

Mice were sacrificed at day 56 post transplantation and skin, lung, liver, spleen tissues were examined for human T/B cell, regulatory T-cell and macrophage engraftment by immunohistochemistry. Significant hCD4^+^ and hCD8^+^ cell infiltration was seen in the lung (CD4;p = 0.0001, CD8;p<0.05, Fig 2C) even in mice with little engraftment in PB (donor1) when compared to mice with CD34^+^ cells. Impaired regulatory T-cell development has been observed in patients with cGvHD[19], thus the number of regulatory T-cell was examined in the target organs by IHC. As expected, little or no hCD4^+^Foxp3^+^ cells (regulatory T-cell) were seen in either the lung or liver (S4 Fig).

Mice with G-hPBMCs from donor 2 had higher engraftment in both the liver and the lungs, (Fig 2C and 2D), especially for hCD8^+^ cells (lung; mean donor 1 12.9%vs. 38.4%(donor 2), p = 0.001, and liver; 2.0%(donor1), 27.8%(donor2), p<0.0001). Little to no hCD20^+^ B cells were found in either the lung (1.3%(donor 1), 0.7%(donor 2), donor 1 vs. Donor 2 p = 0.35), or the liver (0.6%(donor 1), 0.3%(donor 2), p = 0.42). In contrast, hCD20^+^ B cells were found in the spleen of all mice with G-hPBMC (3/3 donor 1, 4/4 donor 2) suggesting hematopoietic recovery. A high percentage of hCD68^+^ macrophages was detected in the lung 0.3%(donor 1), 3.3%(donor 2), p<0.05) and especially in the liver (0.4%(donor 1), 6.4%(donor 2), p<0.0001). In the control mice transplanted with hCD34^+^ cells, no macrophages were detected.

### Lung pathology in NSG mice with G-hPBMCs 56 days post transplantation

**Fig 3:**
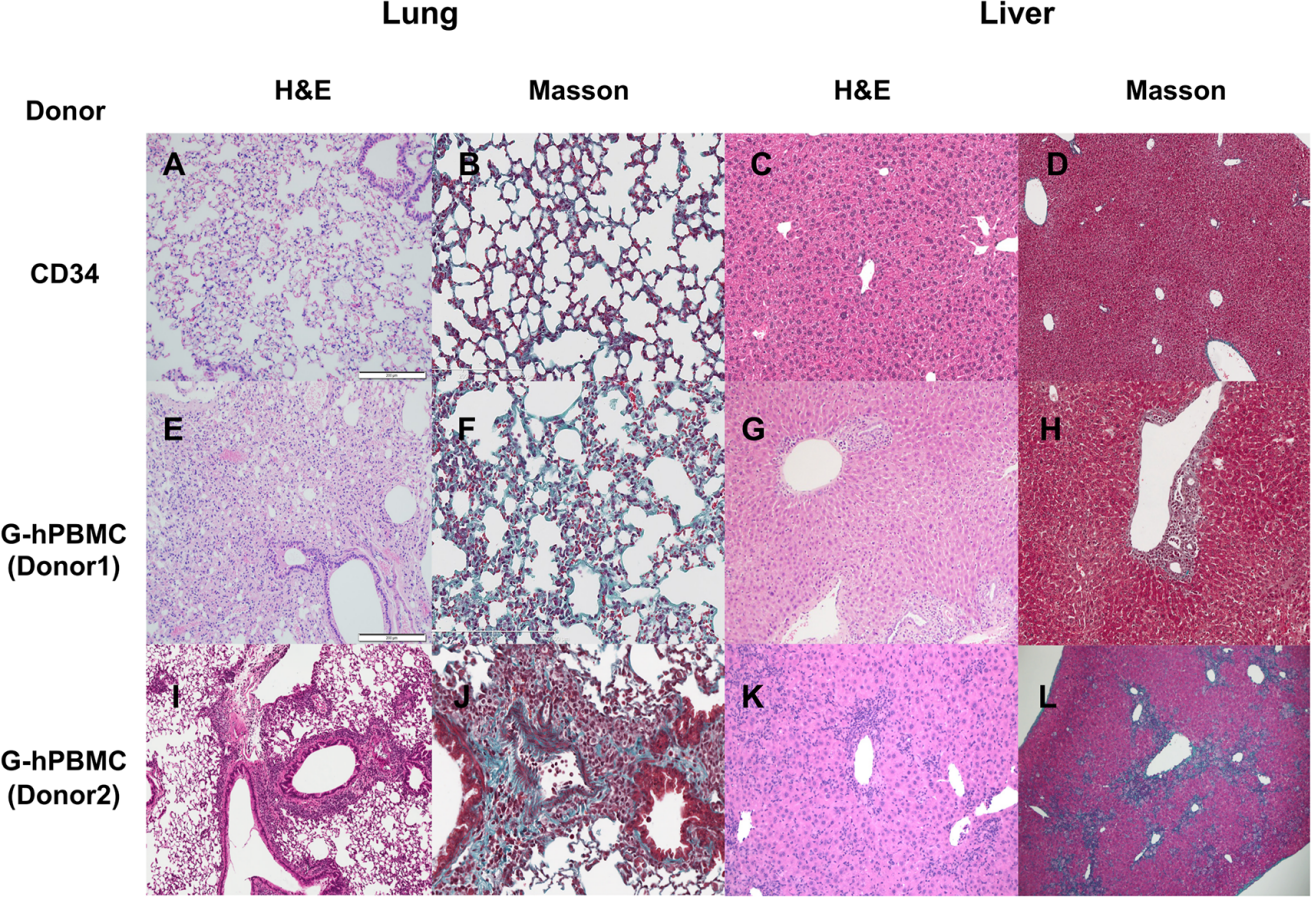
Histopathology in NSG mouse 56 days post transplantation. Lung and liver from mice received CD34+ cells (A, B, C, D) or G-hPBMCs (Donor1; E, F, G, H Donor2; I J, K, L) were stained for H&E (lung/liver 10x) and Masson’s trichrome (lung 20x, liver 10x).

**Fig 4:**
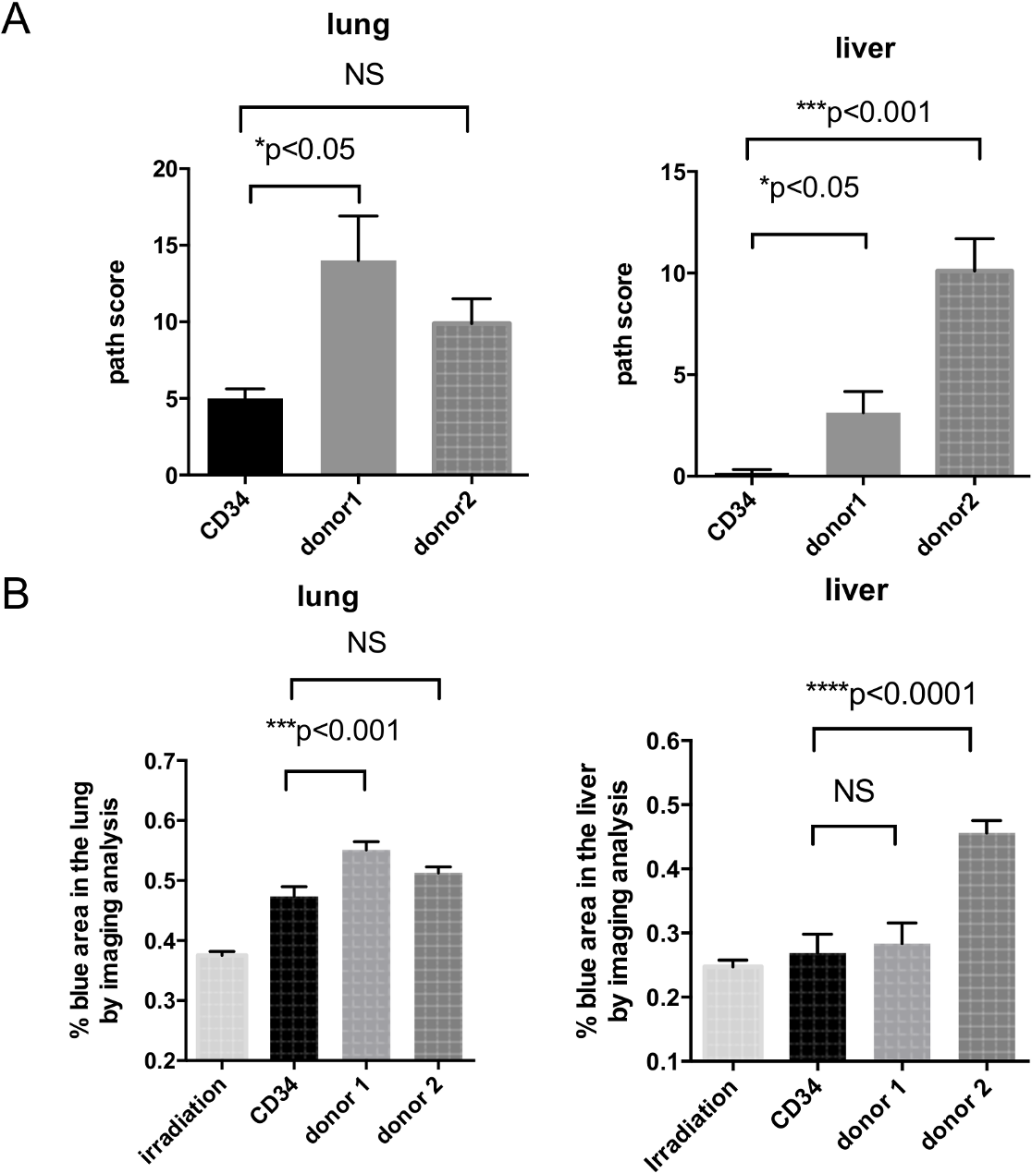
Chronic GvHD pathology score. The pathology of lung, liver and spleen from mice with CD34^+^cells (n = 10) and G-hPBMC (donor 1; n = 8, donor 2; n = 9) were blindly scored 56 days post transplantation (A). A whole slide was scanned and collagen deposition was quantified as a ratio of area of blue staining to total area (B). All results are presented as mean ± SEM.

There were patchy collagen depositions and a few regions of air trapping in the lung from mice with G-hPBMCs (Fig 3E). None of the mice exhibited constrictive bronchiolitis obliterans; however, 5/8 mice (62.5%) in donor 1 and 5/9 mice (55.5%) in donor 2 showed lymphocytic bronchiolitis (S1 Table, Fig 3E), which is considered an early stage of lung GvHD. Mice with donor 1 cells showed more epithelial cell damage, bronchiolar epithelial hyperplasia (75%), alveolar damage (50%) and airway epithelial detachment (62.5%), which may lead to fibrotic change compared to donor 2 (S1 Table). Donor 1 mice also showed a significantly increased pathology score compared to control mice with hCD34^+^cells. (Fig 4A, p<0.05)

The degree of fibrosis in the lung was moderate. Masson's trichrome staining revealed that mice with G-hPBMCs had thickening of alveolar wall in some areas (Fig 3F and 3J). Regions within the parenchyma containing strands of bright eosinophilic material in the septae were interpreted as collagen (fibrosis). Masson's trichrome revealed increased fibrosis in areas of inflammatory cell aggregates.

To support this finding, quantitative image analysis showed that mice with G-hPBMCs had significant increase of fibrosis in the interalveolar septae but not in overall total area compared to control mice with hCD34^+^ cells (p<0.001 Fig 4B).

### Liver pathology in NSG mice with G-hPBMCs 56 days post transplantation

**Fig 5:**
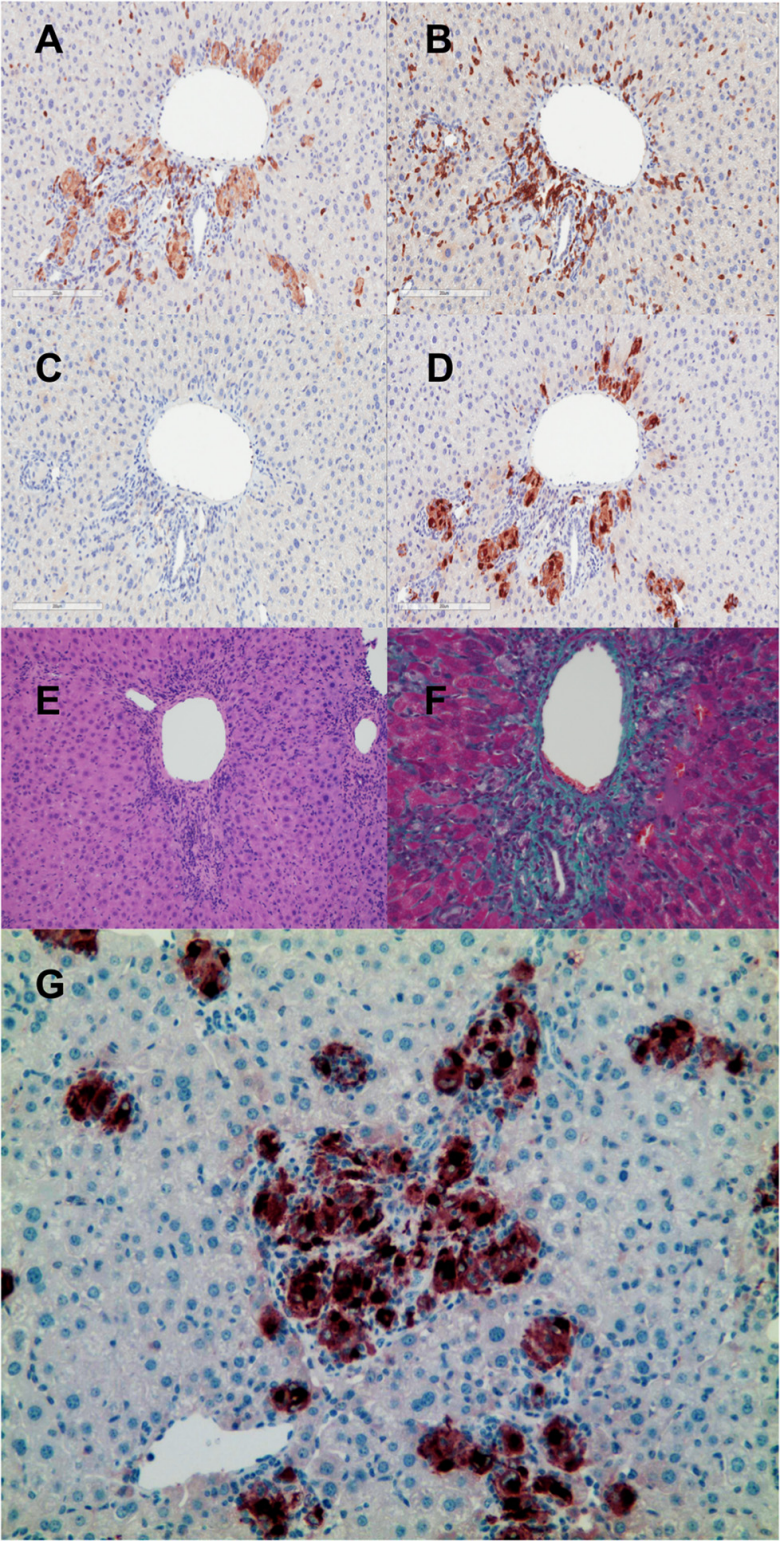
Bile duct inflammation in NSG mice 56 days post transplantation. Liver from mice received G-hPBMCs (donor 2) was stained for hCD4 (A) hCD8 (B) hCD20 (C) hCD68 (D)20x. Histopathology showed ductpenia with lymphocyte infiltration (E; H&E staining 10x), bile duct fibrosis (F; Masson’s trichrome 20x) and macrophage aggregation at bile duct (G, hCD68 positive cells 10x).

Histology showed portal inflammation (4/8 donor 1, 6/9 donor 2), bile duct hyperplasia (2/8 donor1, 9/9 donor 2), bile periductal lymphocyte infiltration (4/8 donor 1, 5/9 donor 2), ductopenia (1/8 donor 1, 5/9 donor 2) and liver cell ballooning (1/8 donor 1, 8/9 donor 2) (S1 Table, Fig 4). Portal fibrosis or bile duct fibrosis was seen in all of the mice with donor 2 cells but in none of the mice with donor 1 cells (0/8 donor1, 9/9 donor2 Fig 4D). The infiltrative cells around the bile ducts and vessels consisted of hCD4^+^, hCD8^+^, hCD68^+^ cells but not CD20^+^ cells (Fig 5A–5D). One of the main histological features of liver GvHD (acute and chronic) is bile duct damage [20]. Similar to human liver GvHD, this model showed bile duct inflammation, ductopenia (Fig 5E) and periductal fibrosis (Fig 5F). Aggregation of human CD68^+^ macrophages was seen around the bile duct together with lymphocytes (Fig 5G).

**Fig 6:**
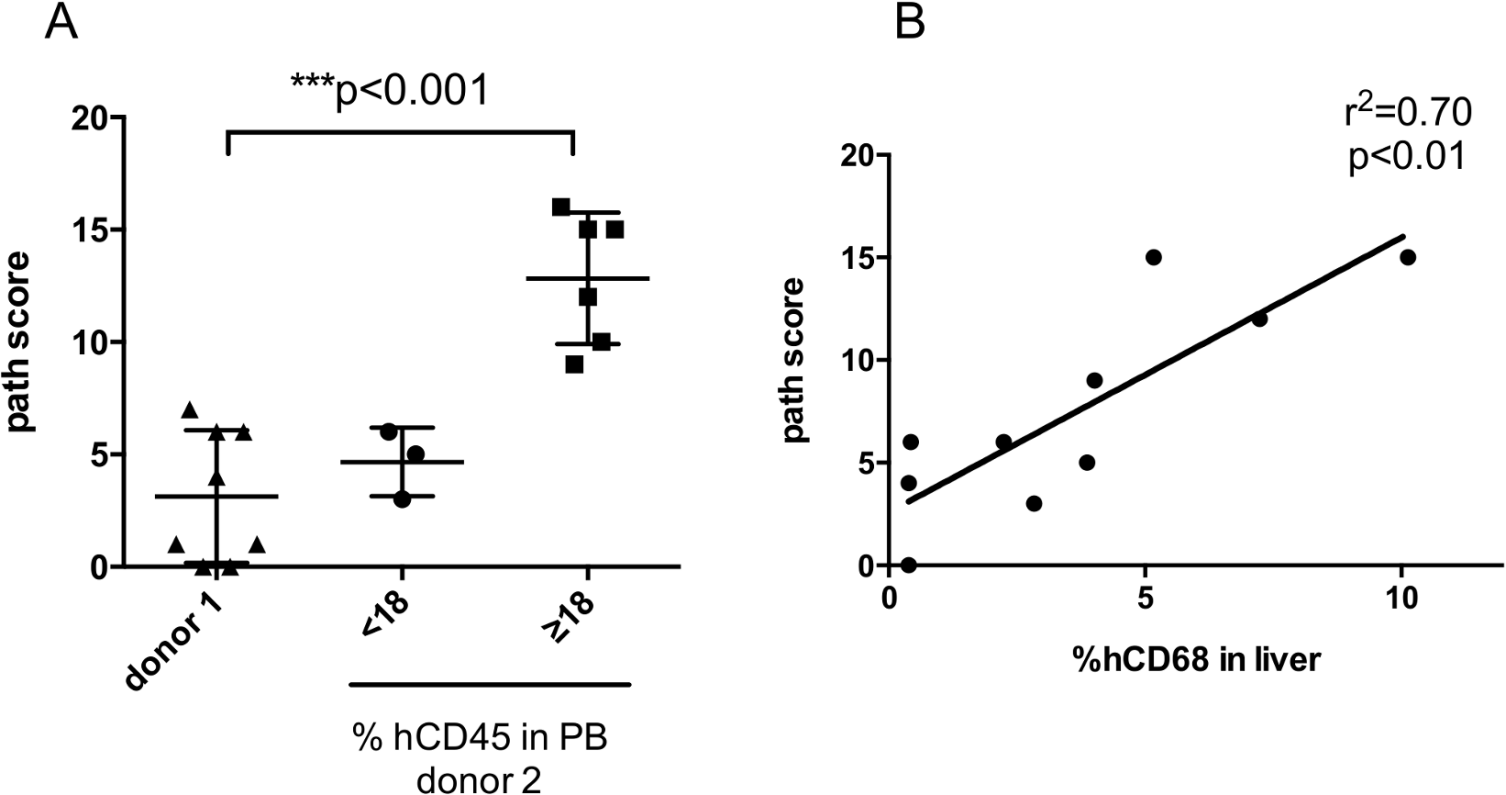
Association between liver pathology and the engraftment level in mice with G-hPBMCs. Liver pathology score from mice with donor 1 (left; n = 8) was compared with donor 2 (right; more than 18% hCD45^+^ cells in PB (n = 6), center; less than 18% in PB (n = 3))(A). Correlation between liver pathology score and % hCD68^+^ cells in liver was analyzed by linear regression (donor 1 and 2; n = 10) (B).

Next we investigated whether the liver pathology was correlated with the percentage of hCD45^+^ cells or the macrophage engraftment level. Mice receiving donor 2 G-hPBMCs had generally a higher level of engrafted hCD45^+^ cells (n = 9, mean±SE: 38.4±10.3%) compared to donor 1 (n = 8, mean±SE: 5.8±3.4%). Three mice receiving donor 2 had less than 18% of hCD45^+^ cells in PB 56 days post transplantation (mean±SE: 7.4±2.3%). They displayed a similar pathology score (median 5, range 3–6) to donor 1 (n = 8, median 2.5, range 0–7), suggesting that liver damage requires higher engraftment levels but was not donor specific (Fig 6A).

### Macrophage infiltration correlates with the liver pathology score

We observed mainly hCD68^+^ cells in the liver of donor 2 mice, but not in donor 1 mice. There was an association between liver pathology score and % of hCD68^+^ cells (Fig 6B, linear regression; r^2^ = 0.70, p<0.01), suggesting that macrophages contributed to disease development. To exclude cross reactivity with mouse macrophages, liver sections were stained with an anti mouse F4/80 antibody, but only Kupffer cells stained positive (S5 Fig).

### Histopathology of other organs from mice with G-hPBMC 56 days post transplantation

The spleen from mice with G-hPBMCs showed very minimal changes compared to control mice. Some mice showed extra medullary hematopoiesis in the subcapsular regions. Skin was unaffected in the epidermal, dermal and subdermal regions. There was no evidence of apoptotic keratinocytes, and very little to absent infiltration of lymphocytes in the skin. The changes were not significant in the GI tract (duodenum, jejunum, ileum, cecum and colon).

### Impaired healing process in organs beyond 56 days post transplantation

**Fig 7:**
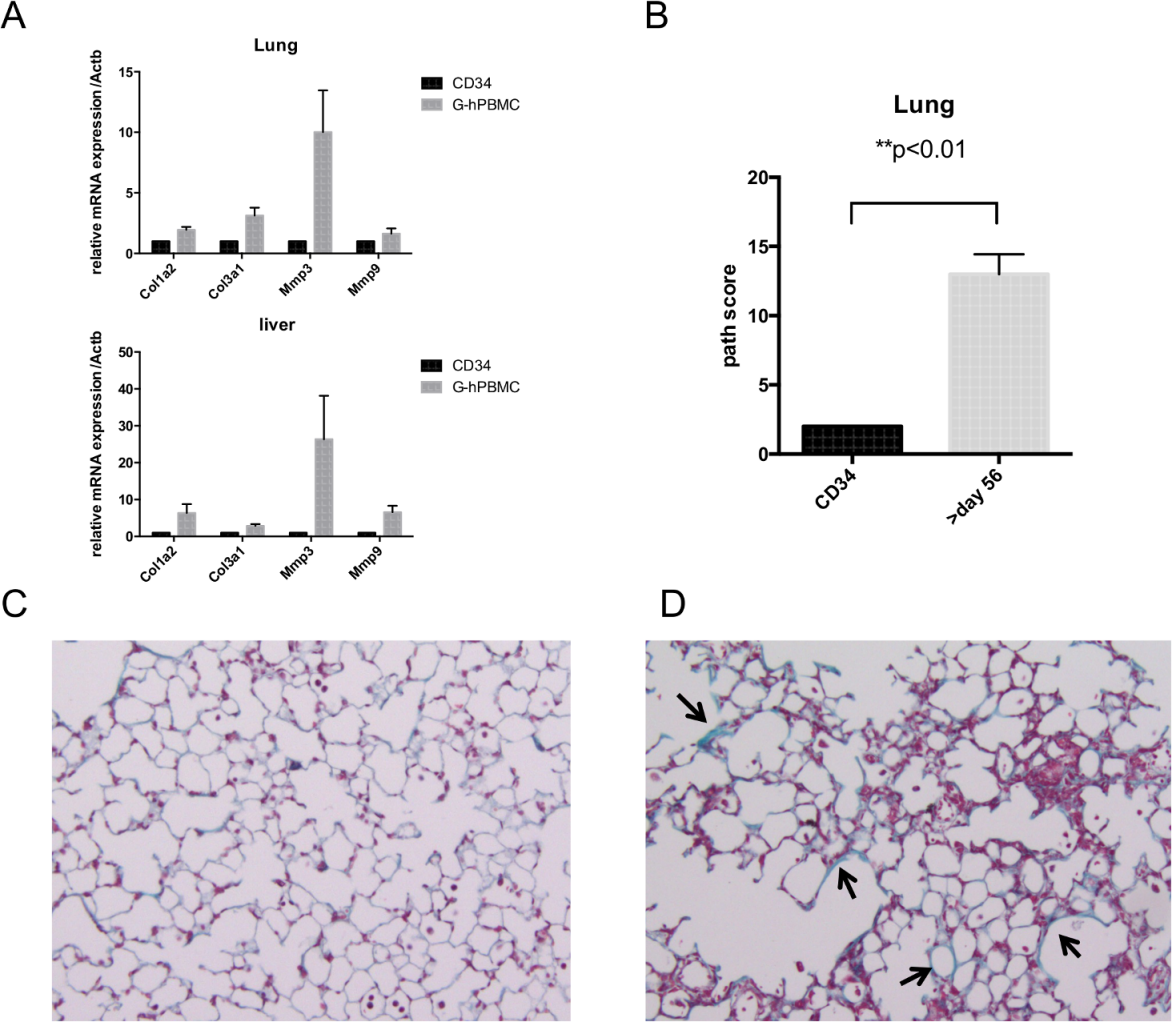
Healing process beyond 56 days post transplantation. Lung / liver tissues were taken from mice receiving CD34 (n = 2) and donor 2 G-hPBMCs (n = 7) 56 days post transplantation. The relative expression of mRNA is shown compared to mouse beta actin. (A). Lung from mice receiving CD34+ cells (n = 6) or donor 2 G-hPBMCs (n = 6) were obtained between day 56 and 84 post transplantation and stained for H&E and Masson’s trichrome (20x). The lung pathology from mice with donor 2 G-hPBMCs (white bar, n = 6) was blindly scored and compared with the lung from mice with CD34^+^cells (black bar, n = 5. One lung was removed due to insufficient size for analysis). All results are presented as mean ± SEM (B). Representative Masson’s trichrome from one of each mouse receiving CD34^+^cells (C) and G-hPBMCs (D) is shown. The arrow indicates thickened alveolar wall. Total of 15 mice (G-hPBMC n = 9, CD34^+^ cells n = 6) were examined in two separate transplantations.

NSG mice that received donor 2 G-hPBMCs did not show lung fibrosis compared to donor 1 at 56 days post transplantation. Matrix metalloproteinase (MMP) is a family of extracellular matrix degradation enzymes, and is expressed by injured tissues. MMP-3 is detectable prior to tissue activation right after injury, whereas MMP-9 is found in active fibrosis. To evaluate the stage of fibrosis, tissue MMP-3 and MMP-9 as well as collagen mRNA were quantified by qRT-PCR 56 days post transplantation. The lung from mice with donor 2 G-hPBMCs, type 1 and type 3 collagen, MMP-3 mRNA expressions were increased compared to CD34^+^cells, but not for MMP-9 mRNA suggesting early-phase fibrogenesis. A similar pattern was observed in the liver (Fig 7A).

To test whether lung remodelling was impaired, NSG mice that received donor 2 G-hPBMCs (n = 9) were kept until 84 days post transplantation to determine the degree of fibrosis. As shown in S6 Fig, three mice with high engraftment died earlier compared to the previous experiment due to rapid expansion of donor cells. Four of nine mice receiving G-hPBMCs died between day 56 and day 84 (endpoint) and two mice survived until day 84. In contrast to the earlier result (Fig 4, S1 Table), the 6 mice that survived more than 56 days had significantly increased lung pathology scores compared to control mice with CD34^+^ cells (Fig 7B, p<0.01, mean±SE;13±1.4) and importantly all 6 mice showed lung fibrosis (Fig 7D) as well as liver fibrosis. This suggests that lung cGvHD can occur at least by using either donor 1 or 2, due to impaired remodelling. Skin fibrosis was also observed in two of four mice that survived more than 56 days (day 67 and 74), whereas mice that died earlier than d56 had no skin GVHD (0 / 4 mice) despite the higher number of engraftment of hCD45^+^cells (S7 Fig). This suggests that the model requires more time to develop skin cGVHD, and possibly the duration of inflammation may influence the severity of target organs.

### Dose adjustment of mature T and CD34+ cells is required for sustained engraftment and inducing fibrosis

Initially donor 3 G-hPBMCs failed engraftment possibly due to lower viability in contrast to the other 2 donors. It was hypothesized that the number of viable mature T cells as well as CD34^+^ cells should be adjusted for each donor. To address the threshold in order to achieve durable engraftment in the model, we have added 6 mice (in 2 experiments) using donor 3 G-hPBMCs at a dose of 2x10^6^, which contain a greater number of viable CD3^+^T (2.6x10^5^) and approximately the same number of viable CD34^+^ cells (2.2x10^4^) in line to that of donor 2. Mice were followed until 56 days post transplantation and the histopathology was examined.

All mice survived (6/6 mice), and the engraftment of hCD45^+^ cells in PB was significantly improved at the mean of 8.5±4.7(SE) %. Histopathology showed both lung fibrosis (4/6) and liver fibrosis (5/6 mice), which confirms the consistency of cGvHD with lung and liver fibrosis induced by a lower dose of G-hPBMCs in donor 1 and 2. This suggests that mice require a threshold of at least 1.5–2.0 x10^5^ as viable CD3^+^T cells to achieve sustained T cell engraftment, and non-T cell compartment may also help engraftment level.

## Discussion

Liver and lung cGvHD especially with fibrosis are often refractory to treatment. Although fibrosis occurs during the tissue healing process, the extensive fibrosis seen at the late stage of cGvHD as a result of excessive deposition of extracellular matrix often leads to high mortality. Currently, use of anti-fibrotic agents have not been shown to improve survival in patients with cGvHD[21–23]. The role of CD4^+^ T cells in fibrosis has been elucidated in a drug-induced lung fibrosis model. There is increasing evidence that macrophages and fibroblasts are activated by CD4^+^ T cells and regulate fibrosis. The role of macrophages in controlling fibrosis depends on the stage of the healing process[24]. However, the contribution of macrophages to the development of cGvHD is not well described due to a lack of an appropriate model.

For the first time, we demonstrate that NSG mice conditioned with cyclophosphamide and TBI with a low dose of G-hPBMCs leads to chronic lung and liver inflammation with fibrosis. Importantly, these pathological features resemble the histologic findings of human cGvHD. In the lung, we observed multiple cGvHD features including collagen deposition, air trapping, lymphocytic bronchiolitis and fibrosis. Although there is no clear pathological criteria to distinguish acute from chronic liver GvHD, the NIH consensus report for histopathological diagnosis in cGvHD described that ductopenia, portal fibrosis and chronic cholestasis are more common in cGvHD[25], suggesting that our model shows chronic, not acute, liver pathology.

We observed cGvHD pathological change mainly in lung, liver and at a lower level in the gut or skin. G-hPBMCs in RAG2-/- γc-/- mice exhibit high levels of engraftment in lung, liver and spleen, but low engraftment in gut and skin [9], suggesting that lung and liver are likely to be target organs by G-hPBMCs, which is consistent with the clinical observation with increased incidence of lung cGvHD post peripheral blood stem cell transplantation. Although our model did not have significant gut pathology, the severe human clinical manifestations occur in lung and liver, demonstrating the importance of our cGvHD model with fibrosis.

Thus far, two humanized cGvHD mouse models have been reported; created by transplanting fetal liver and thymus into NSG mice (BLT mice), which allows for the expansion of multiple lineages of cells from CD34^+^ cells in the fetal liver or cord blood [13,14]. These mice present with symptoms of scleroderma and multiple organ damage, occurring more than 120 days post transplantation without causing aGvHD. Given that aGvHD is one of the major risk factors for cGvHD, this would suggest that T cells play a prominent role in the development of cGvHD. Since the previous xenograft mouse model with adoptive transfer of mature T cells is associated with the development of aGvHD with high mortality rate except 1 out of 30 mice with skin cGvHD, [9], the high incidence of cGvHD in this model suggests that chronic inflammation driven by the combination of macrophages and mature T cells from the graft results in fibrosis. Yet the data cannot exclude possibilities that some of the cGVHD manifestations in the recipients may be the result of the transfer of mature T-cells along with the graft. Thus further studies are needed to distinguish if this is a model of disease due to adoptive transfer of mature T cells or mediated by engrafted cells generated in the host, or both.

Macrophages have a bidirectional role in inflammation, and are classified as M1 and M2 macrophages. Increased numbers of infiltrating macrophages in tissue has been observed in refractory aGvHD[26] and cGvHD[27,28] patients. However, it remains unclear whether macrophages promote or inhibit cGvHD. Our pathological findings demonstrated that CD68^+^ macrophages infiltrate into inflammatory sites such as the bile ducts. Since there were little or no B cells in the organs, human macrophages are likely to play a role as donor antigen presenting cells (APCs) to promote T-cell activation, which may lead to fibrosis. We noted an association between the number of hCD68^+^ macrophages and pathology score. This may explain the smaller amount of liver fibrosis in mice that received donor 1 cells, where little macrophages were observed. Moreover, the macrophages aggregated where fibrosis occurred, such as the bile duct, suggesting the role of macrophages as APCs that promote T-cell driven fibrosis. Further studies on macrophage function in cGvHD and fibrogenesis are currently under way.

The range of engraftment of hCD45^+^ cells needs further evaluation. Lockrige *et al*. showed that hCD68^+^cells in the liver were generated from CD34^+^cells, causing liver fibrosis. The higher percentage of CD34^+^ cells in donor 2 (S2 Fig) may be associated with higher engraftment level of hCD68^+^ cells. One and five million G-hPBMCs were initially tested, and it was clear that engraftment was dose dependent. Subsequently, 1x10^6^ G-hPBMCs from donor 1 and donor 2 successfully induced cGvHD without causing aGvHD, whereas donor 3 required 2x10^6^ G-hPBMCs. Dose adjustments based on viable cell number for both CD3^+^T cells and CD34^+^ cells are required among donors. Although donor 1 G-hPBMCs had the higher number of CD3^+^ T cells than the other, mice showed lower engraftment in the liver. This may have contributed to different homing pattern of donor cells, and therefore the model may serve as a tool to examine donor variability. The mice that survived more than 56 days exhibited more severe cGvHD including the involvement of the skin. Therefore, our model mimicked the early onset of cGvHD due to T-cell and macrophage-driven inflammation as observed in aGvHD. To the best of our knowledge, this is the first paper to show fibrosis by G-hPBMCs in NSG mice.

Lastly, we found that the number of hCD8^+^T cells was increased in organs from cGvHD mice. hCD4^+^ T cells are generally considered to cause cGvHD. However, a recent study found that IL-13 producing CD8^+^ T cells are involved in modulating dermal fibrosis in systemic sclerosisi (SSc) patients [29]. Malard *et al*. showed that cytotoxic CD8^+^ T cells infiltrated the liver in patients with liver cGVHD[30], supporting our humanized liver cGvHD model as an effective tool for investigating the mechanism of graft-versus-leukemia effect in patients with cGvHD. The role of the MHC class I molecule on donor APCs, such as macrophages, in activating CD8^+^ T cells in the humanized cGvHD model requires further investigation.

Our model is relatively easy to establish, and shows faster engraftment than previous reports; 8 to 12 weeks compared to more than 18 weeks. In other words, this model can be utilized not only as a pre-clinical model for cGvHD, but also as a tool to investigate donor variability and the role of human donor T cells / macrophages in fibrosis.

In conclusion, we have shown that NSG mice receiving 1x10^6^ G-hPBMCs post CTX administration and TBI present with lung and liver fibrosis post transplantation. Human CD4/8^+^ T cells and human CD68^+^ macrophages play major roles in disease development. Further investigation is needed to unravel the mechanism of fibrotic change in patients with cGvHD, with the prospect of looking for a pathophysiologically driven therapy that may lead to a better outcome for patients with this devastating side effect of stem cell transplantation.

## Supporting Information

S1 Table The modified cGvHD pathology scale in NSG mice from 2 donors. The list shows all categories used for the modified pathology score and the number shows positivity per total number analyzed for each donor.
(TIF)

S1 Fig Survival in NSG mice after receiving different dose of G-hPBMCs. G-hPBMCs were injected into NSG mice after 200cGy TBI at 1x10^6^, 5x10^6^, 10x10^6^, 20x10^6^ cells and monitored survival for 56 days post transplantation compared to control (irradiation only).
(TIF)

S2 Fig Viability of CD3^+^ T cells and CD34^+^ cells from each donors. Live cell count was analyzed by flow cytometry as fixable aqua-/Annexin V- cells (live cell) gated on hCD3 or hCD34^+^ cells.
(TIF)

S3 Fig Human platelet recovery in NSG mice post transplantation.Peripheral blood from mice with donor 2 G-hPBMCs was analyzed by flow cytometry 42 days post transplantation (n = 12). All mice showed human platelet recovery (left; one representative, right; % of human platelet in the total platelet).
(TIF)

S4 Fig Negative CD4^+^/Foxp3^+^ cells in the lung and the liver. Lung (A) and Liver (B) were stained for hCD4 (pink) and hFoxp3 (brown). Representative IHC from one of mice with G-hPBMCs (n = 7) is shown.
(TIF)

S5 Fig Mouse F4/80 expression in the liver 56 days post transplantation. Liver from mice receiving CD34^+^ cells (A; n = 3) or G-hPBMCs (B; n = 5) were stained for anti-mouse F4/80 antibody. The red arrows indicate kupffer cells (mF4/80^+^). The black arrows indicate the infiltrating macrophages (mF4/80^-^) near the portal vein.
(TIF)

S6 Fig Long-term survival in NSG mice receiving donor 2 G-hPBMCs. Mice were injected either 1x10^5^ CD34^+^cells (solid line, n = 6) or 1x10^6^ G-hPBMCs (dash line, n = 9) of donor 2 and the survival was monitored until 84 days post transplantation.
(TIF)

S7 Fig Skin fibrosis from mice receiving donor 2 G-hPBMCs. Skin from mice received CD34^+^ cells (A) and donor 2 G-hPBMCs (B) were taken on day 67 at the end point and stained with H&E. The arrows indicate the scleroderma change.
(TIF)
