# Supplementary figures and images for: Humanized Chronic Graft-versus-Host Disease in NOD-SCID il2rγ-/- (NSG) Mice with G-CSF-Mobilized Peripheral Blood Mononuclear Cells following Cyclophosphamide and Total Body Irradiation
Source: PLoS One. 2015 Jul 15;10(7):e0133216. doi: 10.1371/journal.pone.0133216 (PMC4503770; doi:10.1371/journal.pone.0133216)

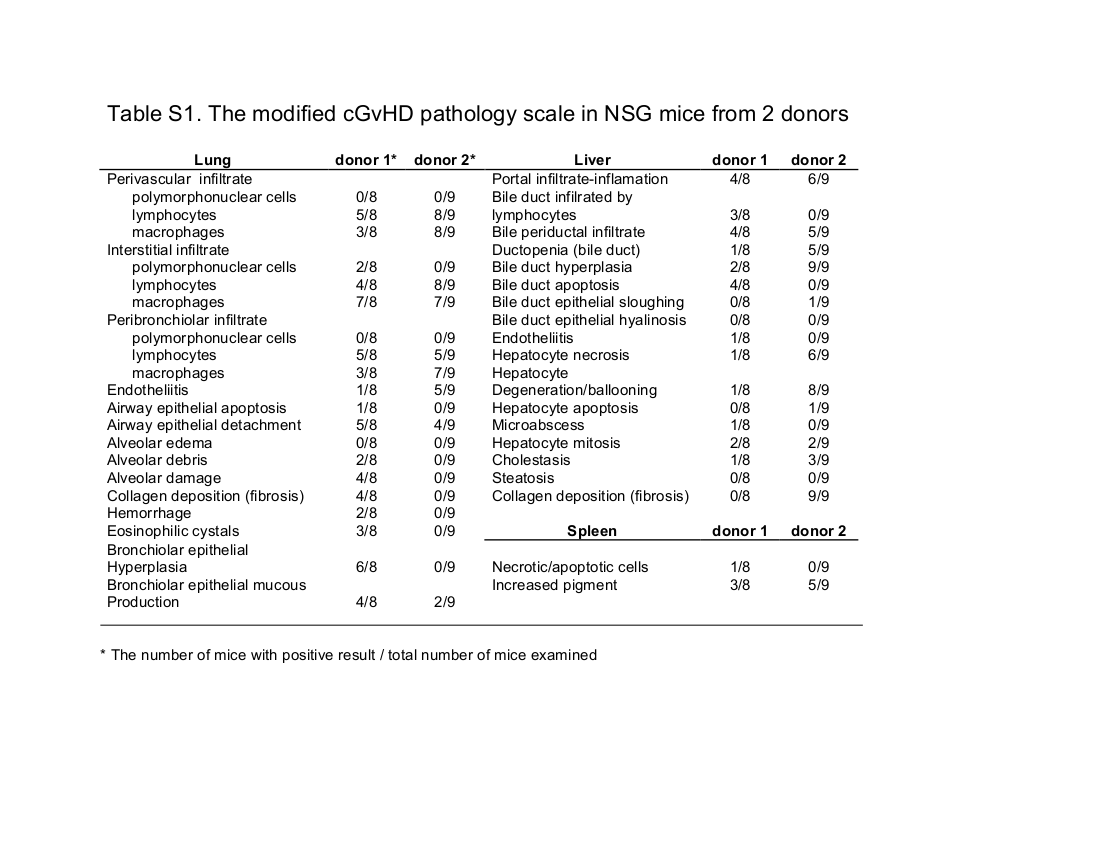

Supplement: S1 Table — The list shows all categories used for the modified pathology score and the number shows positivity per total number analyzed for each donor. (TIF) [file pone.0133216.s001.tif]

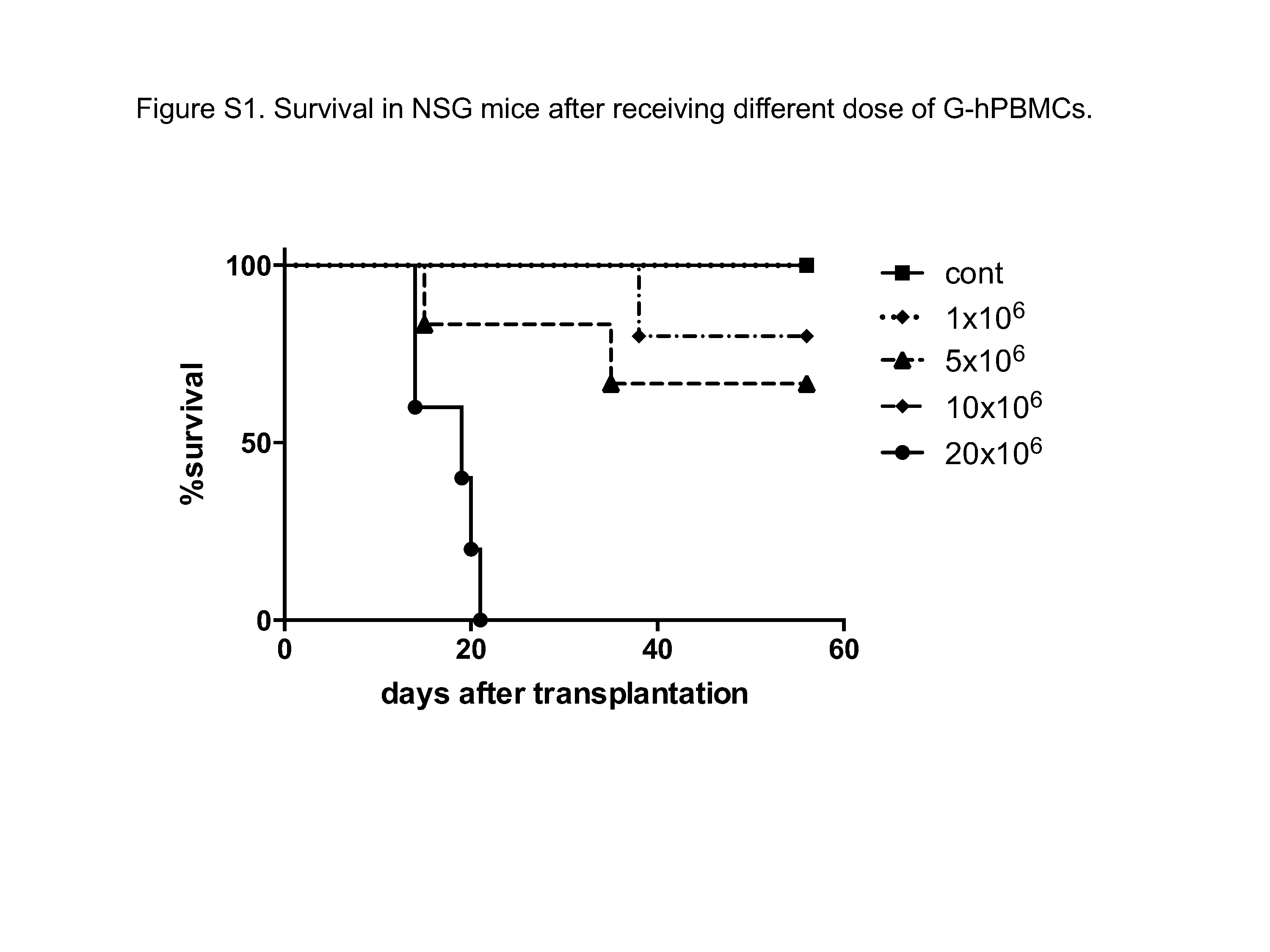

Supplement: S1 Fig — G-hPBMCs were injected into NSG mice after 200cGy TBI at 1x10^6^, 5x10^6^, 10x106, 20x106 cells and monitored survival for 56 days post transplantation compared to control (irradiation only). (TIF) [file pone.0133216.s002.tif]

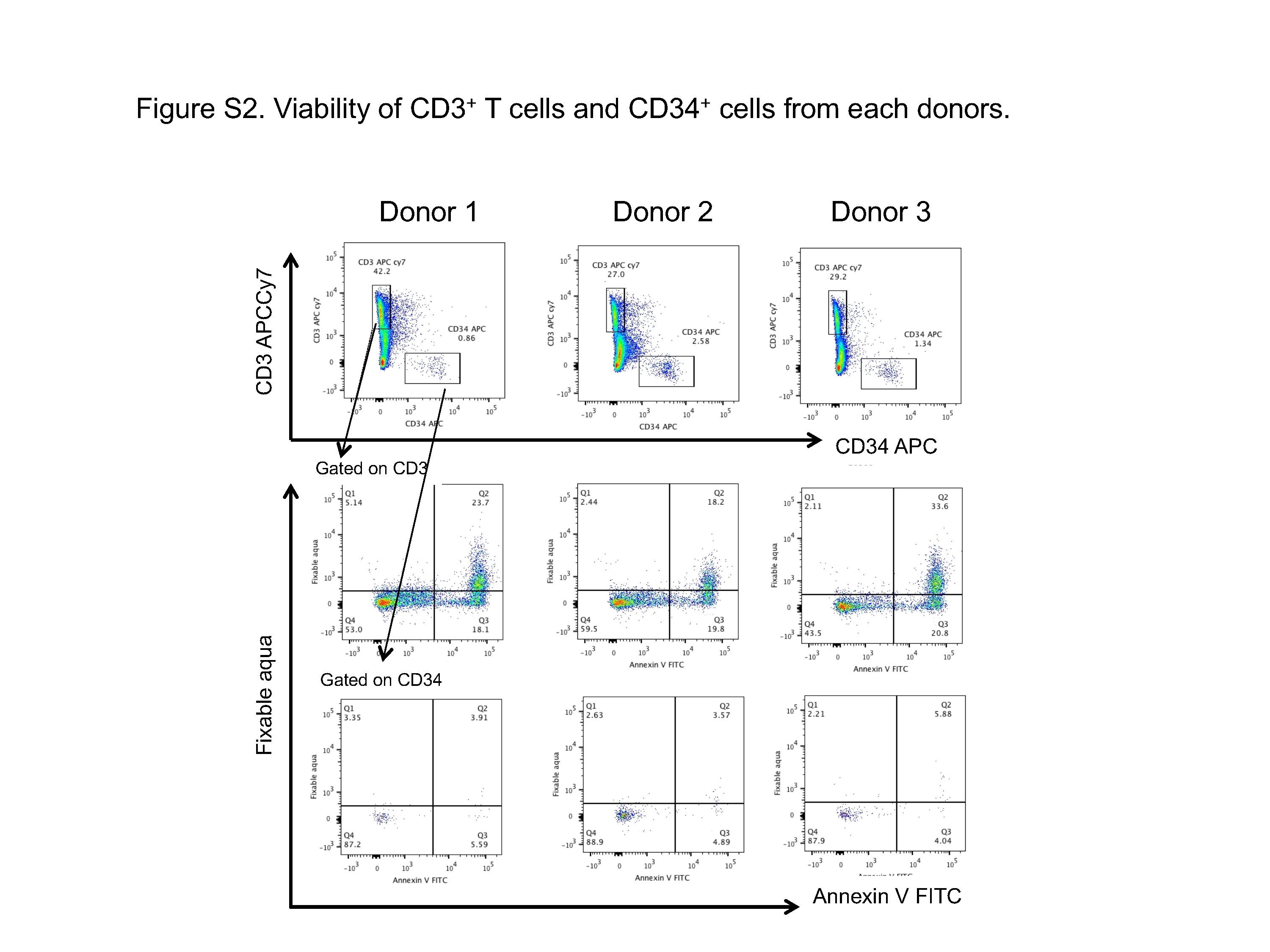

Supplement: S2 Fig — Live cell count was analyzed by flow cytometry as fixable aqua-/Annexin V- cells (live cell) gated on hCD3 or hCD34+ cells. (TIF) [file pone.0133216.s003.tif]

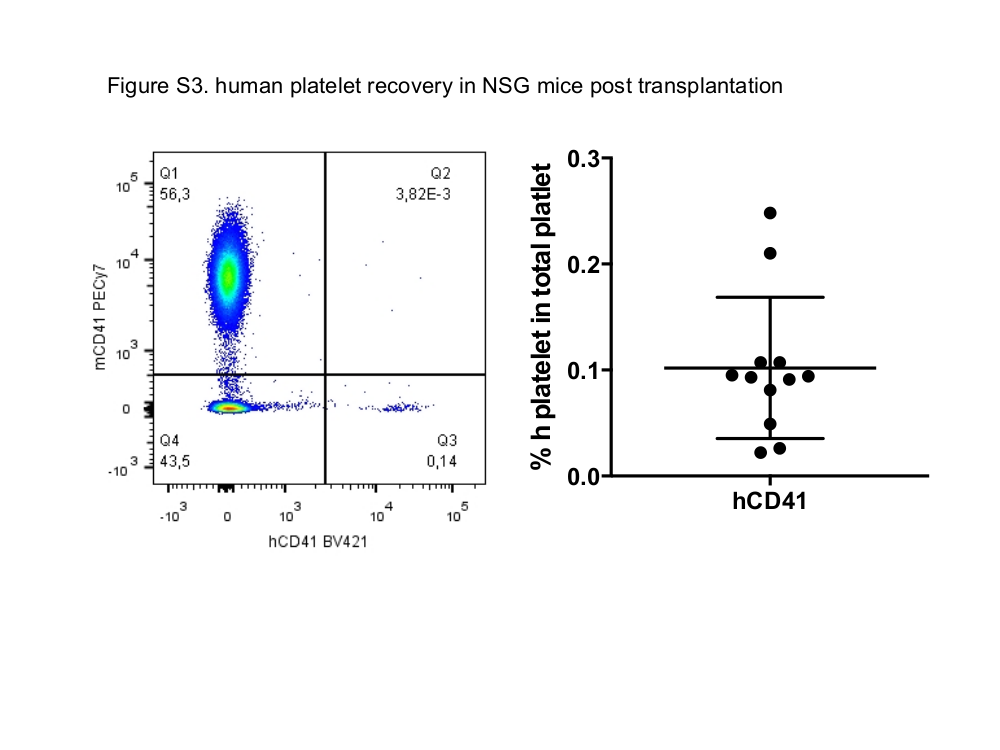

Supplement: S3 Fig — All mice showed human platelet recovery (left; one representative, right; % of human platelet in the total platelet). (TIF) [file pone.0133216.s004.tif]

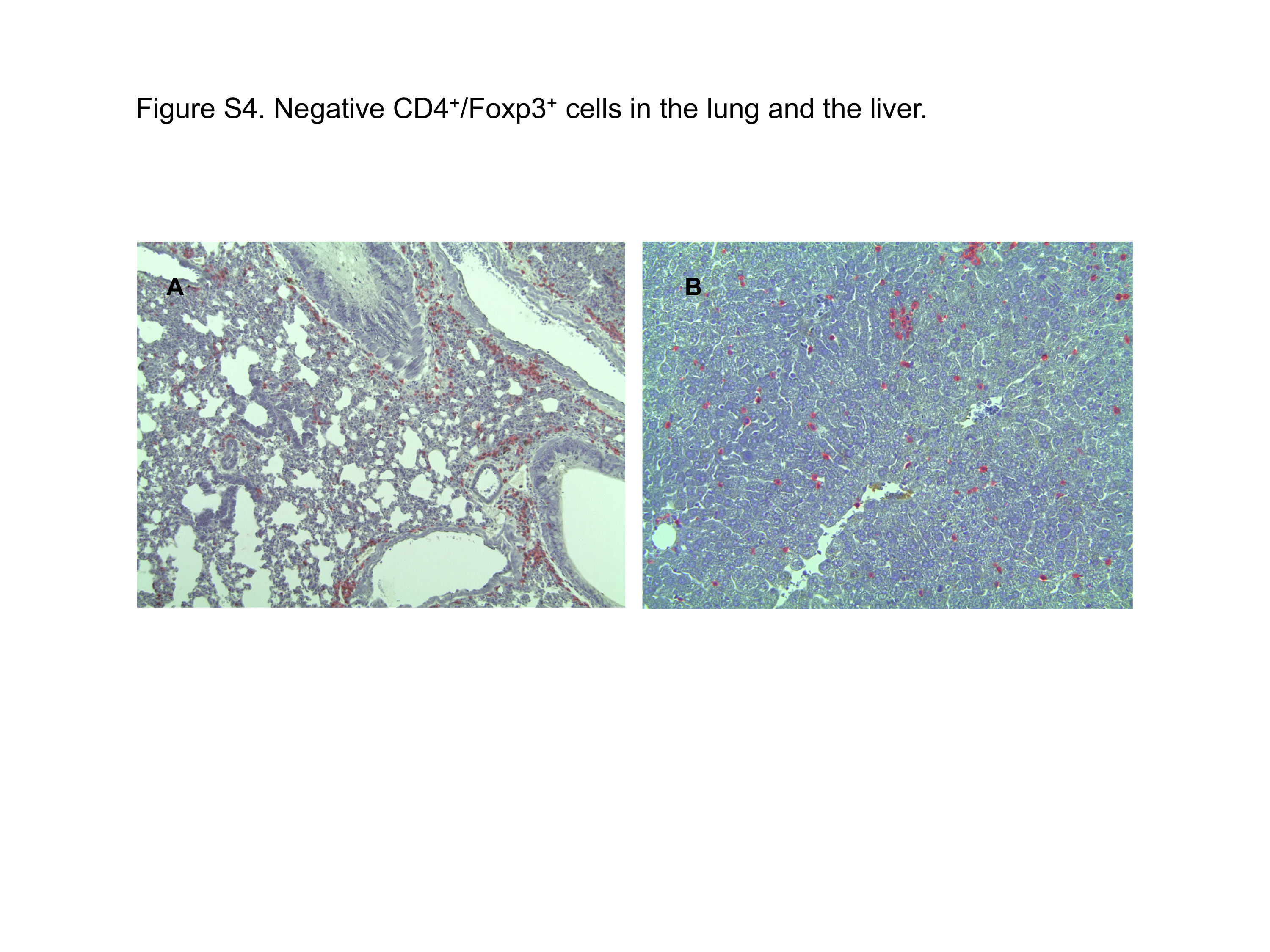

Supplement: S4 Fig — Lung (A) and Liver (B) were stained for hCD4 (pink) and hFoxp3 (brown). Representative IHC from one of mice with G-hPBMCs (n = 7) is shown. (TIF) [file pone.0133216.s005.tif]

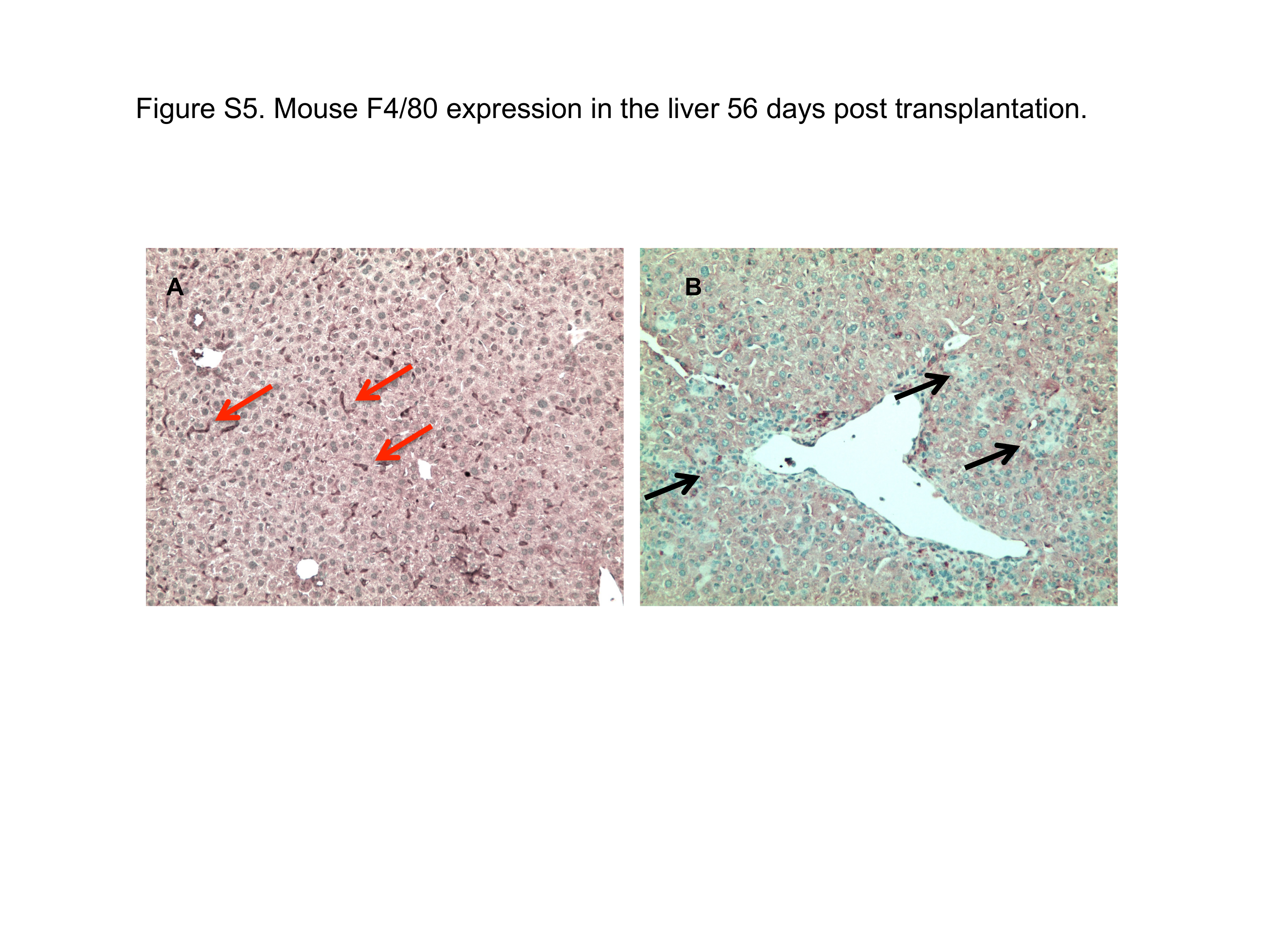

Supplement: S5 Fig — Liver from mice receiving CD34+ cells (A; n = 3) or G-hPBMCs (B; n = 5) were stained for anti-mouse F4/80 antibody. The red arrows indicate kupffer cells (mF4/80+). The black arrows indicate the infiltrating macrophages (mF4/80-) near the portal vein. (TIF) [file pone.0133216.s006.tif]

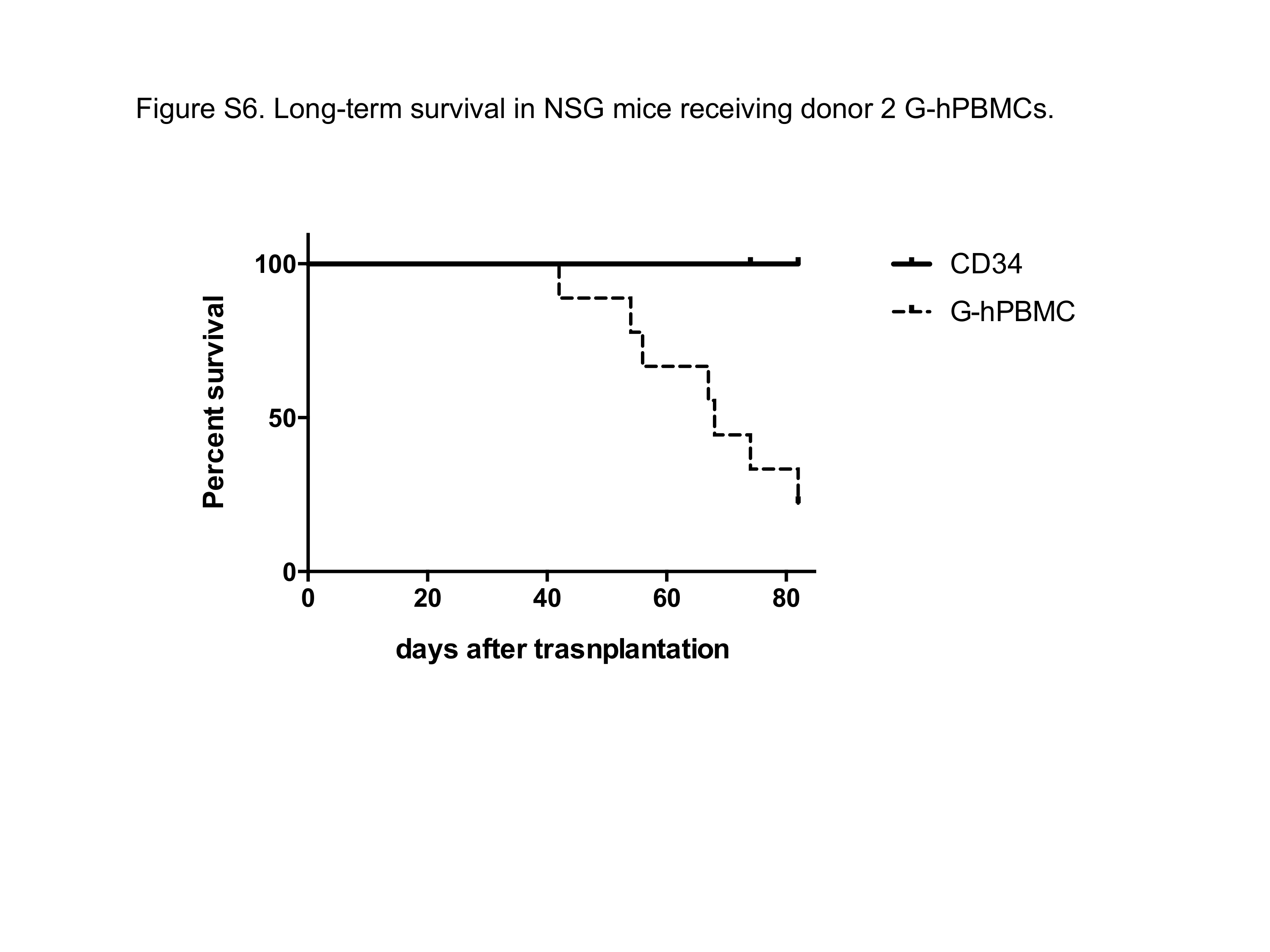

Supplement: S6 Fig — Mice were injected either 1x10^5^ CD34+cells (solid line, n = 6) or 1x10^6^ G-hPBMCs (dash line, n = 9) of donor 2 and the survival was monitored until 84 days post transplantation. (TIF) [file pone.0133216.s007.tif]

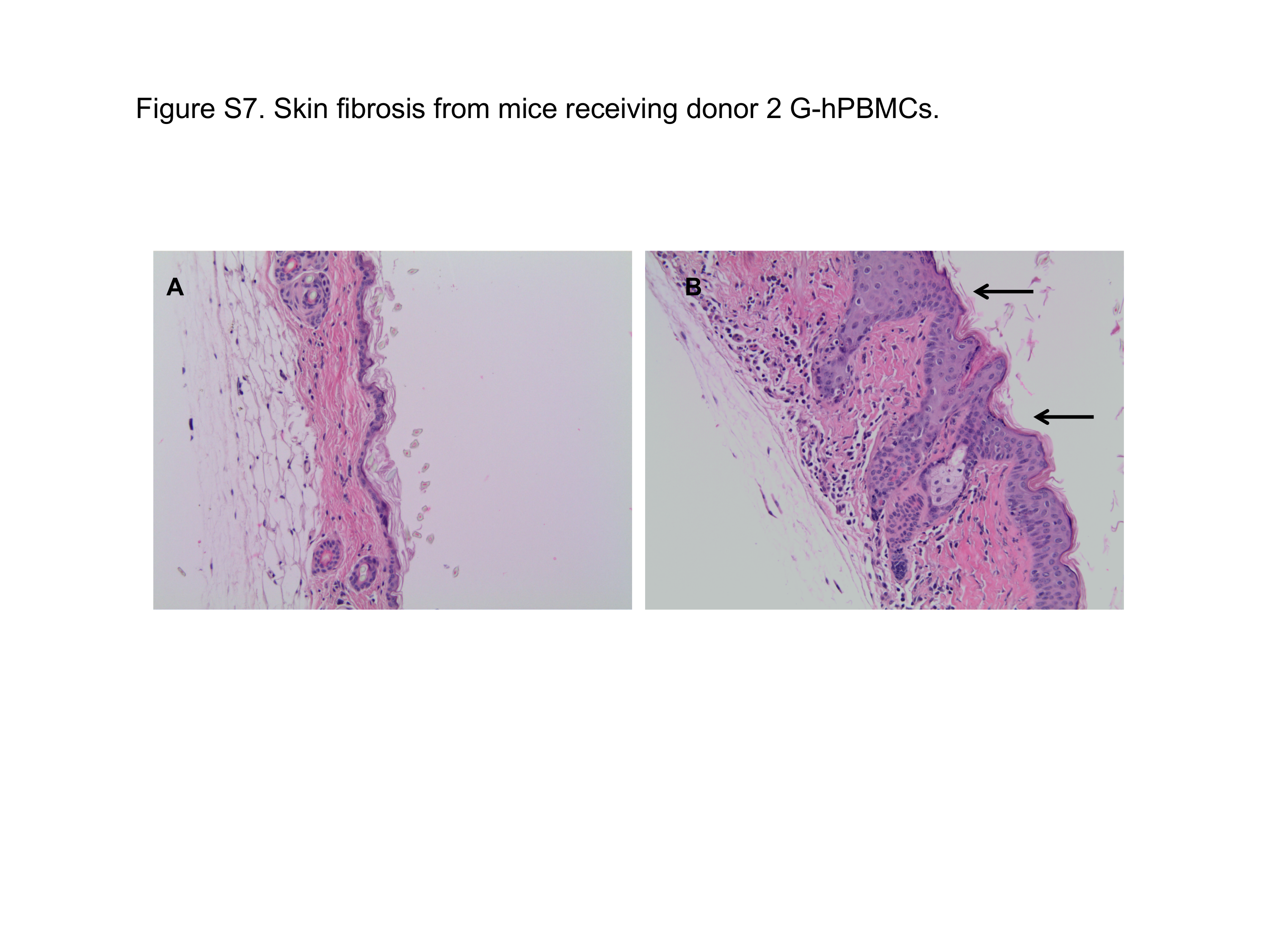

Supplement: S7 Fig — Skin from mice received CD34+ cells (A) and donor 2 G-hPBMCs (B) were taken on day 67 at the end point and stained with H&E. The arrows indicate the scleroderma change. (TIF) [file pone.0133216.s008.tif]
